# Articular Cartilage Tissue Engineering: Cells, Bioinstructive Scaffolds, Immunological Microenvironment, and Emerging Technologies

**DOI:** 10.3390/bioengineering13070795

**Published:** 2026-07-11

**Authors:** Sedeek Mosaid, Yousif Jihad, Mostafa Jihad, Ashok Marudanayagam, Paul Lee

**Affiliations:** 1 United Lincolnshire Teaching Hospitals NHS Trust, Lincoln LN2 5QY, UK; yousif.jihad@nhs.net (Y.J.);; 2 Lancashire Teaching Hospitals NHS Foundation Trust, Preston PR2 9HT, UK

**Keywords:** articular cartilage, tissue engineering, mesenchymal stromal cells, bioinstructive scaffolds, biofabrication, 4D printing, immune microenvironment, CRISPR, artificial intelligence, translational maturity

## Abstract

Focal articular cartilage defects retain limited intrinsic repair capacity owing to the avascular, alymphatic and aneural nature of hyaline cartilage. Marrow-stimulation procedures often generate mechanically inferior fibrocartilage with declining benefit within 2–5 years in larger or high-demand lesions, while matrix-induced autologous chondrocyte implantation (MACI) achieves durable 10-year benefit but remains constrained by two-stage logistics, in vitro dedifferentiation and cost. This review integrates the cellular, biomaterial, biochemical and immunological dimensions of articular cartilage tissue engineering with quantitative benchmarks and a critical reading of failure modes, scalability and regulatory standing. We benchmark MACI against single-stage chondron- and progenitor-based therapies; examine mesenchymal stromal cells (MSCs) from bone marrow, adipose, synovium and the infrapatellar fat pad with a mechanistic dissection of the Wnt/β-catenin, IHH–PTHrP, RUNX2/MEF2C and HIF-1α inputs driving hypertrophic drift; reframe scaffolds as bioinstructive environments delivering mechanical, biochemical and tribological cues, including stimuli-responsive and 4D-printed systems and low-intensity pulsed ultrasound (LIPUS) as a non-invasive adjunct; develop the immunological dialogue between altered native cartilage, the infrapatellar fat pad–synovium unit and engineered constructs; and appraise CRISPR-based cell engineering and artificial-intelligence applications in biofabrication. We classify the principal approaches into four explicit translational tiers so that the evidentiary standing of each strategy is transparent. Translation will be paced by standardised potency assays, immune-aware construct design and robust long-term in vivo evidence.

## 1. Introduction

Articular cartilage covers the diarthrodial surfaces of synovial joints and provides a near-frictionless, load-bearing interface essential for locomotion. Its mechanical performance derives from a hierarchical, anisotropic extracellular matrix (ECM)—water (65–80% wet weight), type II collagen and proteoglycans—populated by chondrocytes occupying approximately 1–2% of tissue volume in adult human articular cartilage [1,2]. The same architecture that confers durability under physiological loads also imposes a profound vulnerability: cartilage is avascular, alymphatic and aneural [3]. Following focal injury, chondrocyte migration beyond the lacunae is severely restricted, and the systemic progenitor recruitment that drives tissue repair in vascularised tissues is absent; intrinsic restorative repair is therefore inadequate [4].

This biological limitation has direct epidemiological and economic consequences. Focal chondral defects are identified in more than 60% of patients undergoing routine knee arthroscopy [5], and biologically unfavourable lesions may contribute to a catabolic intra-articular environment and an increased risk of later osteoarthritic degeneration [6]. The Global Burden of Disease Study 2021 estimated that approximately 595 million people worldwide were living with OA in 2020 (7.6% of the global population) [7]. The knee is the dominant joint subtype, accounting for over 374 million prevalent cases and approximately 12 million disability-adjusted life-years in secondary analyses of the same dataset [8]. In the United Kingdom, the rising volume of total knee arthroplasty—performed predominantly for end-stage OA—is projected to increase by approximately 36.6% to over 137,000 procedures annually by 2060 [9], placing growing pressure on National Health Service surgical capacity and prompting value-based healthcare analyses to safeguard the sustainability of the integrated care pathway [10]. Treatment of symptomatic focal defects aims primarily to improve pain and function; whether current repair strategies reduce the long-term risk of OA remains uncertain and requires longer-term comparative evidence, but the unmet clinical need is well defined.

Marrow stimulation, popularised in the form of microfracture by Steadman and colleagues in the late 1980s, remains a first-line procedure for small (<2 cm^2^) defects because of its technical simplicity and low cost [11]. The repair tissue, however, is frequently dominated by type I collagen and behaves more as fibrocartilage than hyaline cartilage; structural deterioration and declining clinical benefit are commonly reported within 2–5 years, particularly in larger or high-demand lesions, with return-to-sport rates declining progressively after the first post-operative year [12,13]. Cell-based and tissue-engineered approaches were developed to address this limitation. Matrix-induced autologous chondrocyte implantation (MACI), the third-generation form of autologous chondrocyte implantation (ACI), is FDA-approved in the United States (2016) for symptomatic full-thickness knee cartilage defects in adults; in Europe, autologous chondrocyte products fall within the Advanced Therapy Medicinal Product (ATMP) framework under EU Regulation 1394/2007, although MACI’s EU marketing authorisation expired in 2018 after prior suspension. MACI demonstrates sustained patient-reported and structural benefit at minimum 10-year follow-up for appropriately selected defects [14,15], establishing a principal regulatory and clinical benchmark in jurisdictions where it remains approved and reimbursed.

This review examines articular cartilage tissue engineering through four integrated lenses: (i) the structure and biomechanics that any successful construct must reproduce; (ii) cellular sources—from autologous chondrocytes and chondrons to MSCs and induced pluripotent stem cells (iPSCs)—with strategies to control hypertrophic drift; (iii) scaffolds reframed as bioinstructive microenvironments delivering coordinated mechanical, biochemical and tribological cues; and (iv) the immunological dimension, encompassing the response to pathologically altered native cartilage, the infrapatellar fat pad–synovium unit, and the foreign-body response to engineered constructs. We close with an appraisal of CRISPR-based cell engineering, advanced biofabrication and concrete artificial-intelligence applications, identifying the translational gaps that remain.

## 2. Articular Cartilage: Structure, Composition and Biomechanical Demands

Successful regeneration requires reproducing, at least approximately, the hierarchical organisation that underwrites the mechanical performance of native tissue [3,16]. Three features dictate the engineering targets: ECM composition, zonal architecture, and the non-linear, depth-dependent biomechanical response.

### 2.1. Extracellular Matrix Composition

The ECM comprises water (65–80% wet weight), collagens (10–20%) and proteoglycans (4–7%) [17]. Type II collagen accounts for 90–95% of total collagen and provides tensile strength; minor collagens IX, XI and VI stabilise fibril intersections and mediate chondrocyte–matrix coupling within the pericellular matrix [18]. Aggrecan, the dominant proteoglycan, carries densely sulphated chondroitin- and keratan-sulphate glycosaminoglycan (GAG) chains whose fixed negative charge generates a Donnan osmotic swelling pressure restrained by the collagen network [1,19]. The resulting prestressed composite resists compression. A predominance of type I rather than type II collagen in tissue-engineered constructs may compromise this composite mechanical logic and contribute to inferior hyaline-like function and durability [20].

### 2.2. Zonal Architecture

**Figure 1:**
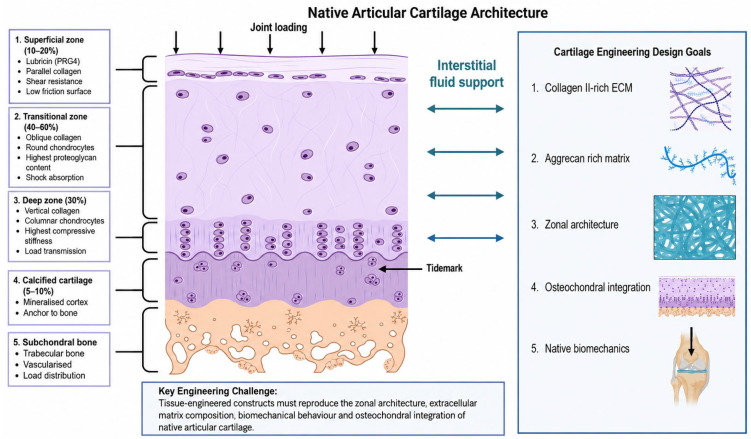
Native articular cartilage architecture and key engineering design targets.

Articular cartilage is partitioned into superficial (tangential), middle (transitional), deep (radial) and calcified zones, each with distinct cell shape, fibre orientation and matrix composition [21]. The superficial zone (≈10–20% of thickness) contains flattened chondrocytes and surface-parallel type II collagen fibrils, providing tensile stiffness and shear resistance; superficial chondrocytes secrete proteoglycan-4 (lubricin/PRG4), the principal boundary lubricant [22,23]. The transitional zone (≈40–60%) features rounded chondrocytes, obliquely arranged fibrils and the highest proteoglycan concentration. The deep zone (≈30%) contains chondrocytes in vertical columns with fibrils oriented perpendicular to the joint surface, conferring the greatest compressive resistance [21]. The calcified zone, separated from uncalcified tissue by the tidemark, anchors cartilage to subchondral bone and mediates gradual force transfer across the osteochondral interface—a graded interface that engineered constructs continue to find difficult to reproduce [24,25]. The structural and biomechanical organisation of native articular cartilage and the corresponding engineering targets are illustrated in Figure 1.

Articular cartilage is organised into superficial, transitional, deep and calcified zones, each characterised by distinct collagen orientation, chondrocyte morphology and extracellular matrix composition. Joint loading is supported through interstitial fluid pressurisation and depth-dependent mechanical properties. Successful tissue-engineered constructs must replicate zonal architecture, collagen II-rich extracellular matrix, aggrecan content, osteochondral integration and native biomechanical behaviour.

### 2.3. Biomechanical Behaviour Beyond the Biphasic Approximation

The classical biphasic poroelastic model describes cartilage as a permeable solid phase (collagen and proteoglycans) saturated by an incompressible fluid phase [26]. Under instantaneous compression, interstitial fluid pressurisation supports up to ~90% of applied load and shields the solid matrix; as load is sustained, fluid exudes through the low-permeability matrix (hydraulic permeability ≈ 10^−15^–10^−16^ m^4^·N^−1^·s^−1^), producing creep until equilibrium is reached, at which point load is borne by the solid ECM and proteoglycan-derived osmotic swelling pressure [27,28].

This framework is necessary but not sufficient. Native cartilage is non-linear, strain-rate-dependent and spatially heterogeneous: stress–strain curves stiffen with strain through progressive collagen-fibre recruitment; the instantaneous (impact) modulus exceeds the equilibrium modulus by one to two orders of magnitude (typical equilibrium 0.5–1.0 MPa, instantaneous approaching 50 MPa); and the equilibrium modulus rises from ~0.3 MPa in the superficial zone to >2 MPa in the deep zone of healthy human femoral condyle [29]. Osteoarthritic degradation reverses this profile—equilibrium modulus declines by 30–60%, hydraulic permeability rises, and the depth-dependent gradient flattens—complicating both modelling and the design of stage-appropriate constructs [29,30]. A poroviscoelastic representation incorporating intrinsic matrix viscoelasticity captures these behaviours more accurately than the biphasic formulation [28].

From these properties, three design constraints follow. Constructs must (i) achieve depth-graded composition and fibre orientation rather than homogeneous fill, (ii) deliver an instantaneous-to-equilibrium modulus ratio compatible with fluid-pressurisation load support, and (iii) integrate stably with calcified cartilage and subchondral bone at the tidemark. Construct success is best judged against these mechanical and structural targets, not by defect-fill volume alone [31,32].

## 3. The Bioengineering Triad: Cells, Bioinstructive Scaffolds, and Bioactive Signals

**Figure 2:**
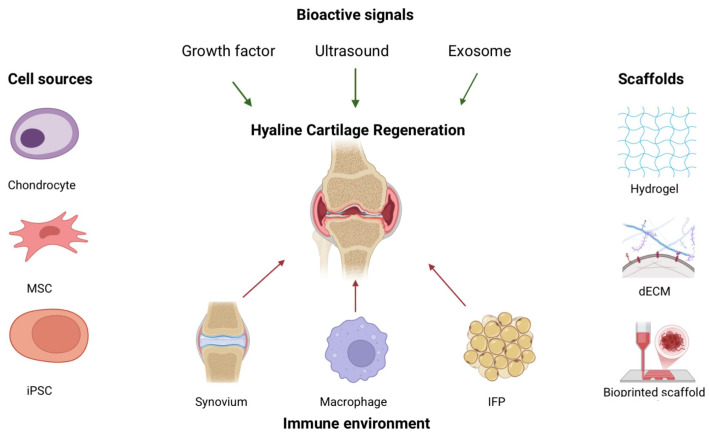
Contemporary bioengineering framework for articular cartilage regeneration. Cartilage tissue engineering is organised around the interaction of cellular sources, bioactive signalling molecules and biomaterial scaffolds. Chondrocytes, MSCs and iPSCs interact with hydrogels, decellularised extracellular matrices (dECMs) and bioprinted scaffolds, while growth factors, extracellular vesicles and ultrasound-mediated stimulation provide instructive biological cues. The immune microenvironment, including synovium, macrophages and the infrapatellar fat pad (IFP), influences regenerative outcomes.

Modern cartilage tissue engineering remains organised by the classical triad—cells, scaffold and bioactive signals—but each component has been substantively reconceptualised. Cell sources are chosen for phenotypic stability rather than expansion potential alone; scaffolds are designed as bioinstructive microenvironments delivering biochemical, mechanical and tribological cues; and bioactive signalling encompasses extracellular vesicles and immune-modulating cues alongside soluble growth factors [15,33]. Cell-free strategies supply the cellular arm through host progenitor recruitment rather than pre-seeded cells (Section 3.3.2). Key characteristics, advantages and limitations of cellular sources are summarised in Table 1. The contemporary bioengineering triad and its interaction with the immune microenvironment are illustrated in Figure 2.

Autologous chondrocytes, mesenchymal stromal cells (MSCs), and induced pluripotent stem cells (iPSCs) represent the primary cellular strategies for cartilage regeneration. While chondrocytes remain the clinical benchmark, stem cell-based approaches offer scalability but introduce challenges related to phenotypic stability, safety, and regulatory translation.

### 3.1. Cellular Sources

**Figure 3:**
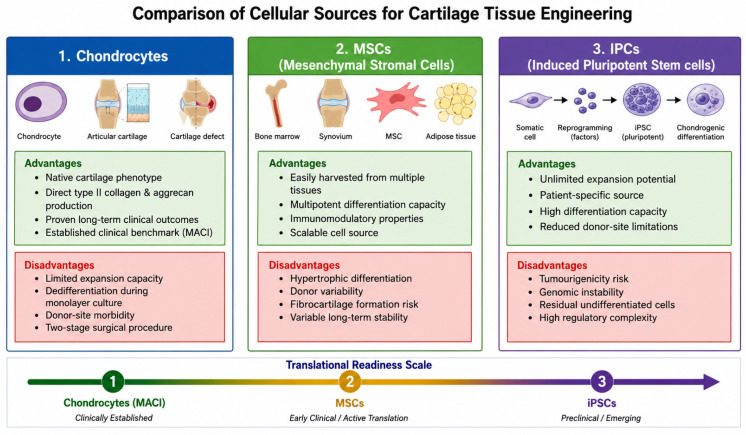
Comparison of major cellular sources for cartilage tissue engineering.

The major cellular sources currently utilised for cartilage tissue engineering, together with their advantages, limitations and translational readiness, are summarised in Figure 3.

#### 3.1.1. Autologous Chondrocytes and Single-Stage Therapies

ACI has progressed from periosteum-covered first-generation procedures to MACI, in which expanded autologous chondrocytes are seeded on a type I/III collagen membrane [34]. Long-term comparative evidence supports MACI: in the SUMMIT randomised trial, MACI achieved a higher responder rate than microfracture at two years (87.5% vs. 68.1%, defined as ≥10-point improvement in both KOOS pain and function) [35], with significantly greater improvements in pain and function maintained at five-year extension follow-up [36]. Systematic review of minimum 10-year outcomes confirms sustained patient-reported benefit and structural durability [15]. Limitations remain real: a two-stage procedure, a regulated period of monolayer expansion during which chondrocyte dedifferentiation can occur, and substantial cost [37].

Single-stage chondrocyte-based therapies mitigate these limitations through intra-operative harvest, processing and immediate reimplantation, eliminating prolonged expansion. Approaches include minimally manipulated autologous chondrocytes, chondron-based constructs (with the pericellular matrix preserved to maintain chondrogenic phenotype) and co-implantation of chondrons with bone-marrow- or synovium-derived stromal cells in hyaluronan or fibrin [38,39]. The first-in-human IMPACT trial showed that one-stage repair with allogeneic MSCs mixed with recycled autologous chondrons is safe and feasible, with clinically meaningful patient-reported improvements [40]; a recent systematic review of co-implantation strategies reports broadly favourable in vivo and early clinical results, although randomised long-term comparators against MACI remain lacking [41]. This reflects a broader paradigm shift from implanted cells as engrafting building blocks toward sources of paracrine and immunomodulatory signals that orchestrate repair by host cells [42].

#### 3.1.2. Mesenchymal Stromal Cells: Source Selection and the Hypertrophy Problem

Mesenchymal stromal cells (MSCs)—bone-marrow- (BM-MSC), adipose- (ASC), synovium- (SM-MSC), umbilical-cord- and infrapatellar-fat-pad-derived (IFP-MSC)—share the International Society for Cellular Therapy (ISCT) minimum criteria (CD73^+^, CD90^+^, CD105^+^; CD45^−^, CD34^−^, CD14^−^ or CD11b^−^, CD79α^−^ or CD19^−^, HLA-DR^−^; plastic adherence with trilineage differentiation potential) but differ substantially in chondrogenic competence, hypertrophic propensity and accessibility [43,44]. SM-MSCs and IFP-MSCs derive from tissues developmentally and anatomically continuous with cartilage and demonstrate consistently superior chondrogenic output in side-by-side comparisons [43]. The dominant translational obstacle is hypertrophic drift: under standard TGF-β-driven chondrogenic protocols, MSC-derived chondrocytes upregulate COL10A1, MMP13, RUNX2 and alkaline phosphatase, calcify and undergo endochondral ossification rather than maintain a stable hyaline phenotype [45].

Mechanistically, this drift recapitulates the embryonic growth-plate programme rather than reflecting a passive culture artefact, which likely explains its resistance to in vitro suppression. Sustained TGF-β/SMAD2/3 input is permissive for chondrogenic induction but insufficient to lock cells in the resting articular phenotype, and several hypertrophy-driving inputs run in parallel. Canonical Wnt/β-catenin signalling, activated by loading and supra-optimal matrix stiffness, cooperates with RUNX2 to upregulate COL10A1, MMP13 and VEGF. The IHH–PTHrP feedback loop that in vivo confines hypertrophy to the deep growth-plate zone is partially uncoupled in MSC cultures because PTHrP autocrine output is low, allowing IHH-driven RUNX2 and MEF2C to dominate. p38 and ERK1/2 MAP-kinase activity, sensitive to BMP and FGF inputs, further reinforces RUNX2, while loss of SOX9-mediated repression of COL10A1 removes a key brake. Hypoxia preserves the articular phenotype through HIF-1α-mediated RUNX2 suppression and SOX9 stabilisation, but implanted constructs re-equilibrate with synovial oxygen tensions higher than the 2–5% used in preconditioning [43,45].

**Figure 4:**
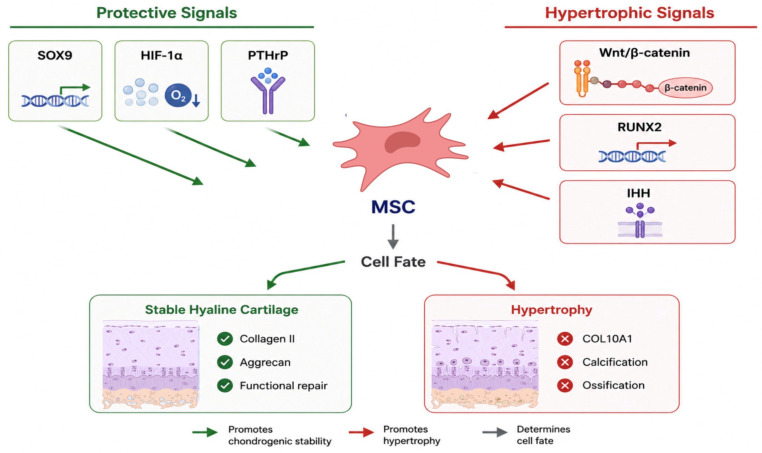
Molecular regulation of MSC fate during chondrogenic differentiation.

Strategies to suppress hypertrophy include hypoxic preconditioning (2–5% O_2_), combined TGF-β3 plus BMP-6 with PTHrP signalling, mechanical preconditioning, and matrix-mediated cues reproducing the low-stiffness, high-water-content niche of the superficial and middle zones [43,45]. Each addresses a single node of the network—PTHrP for IHH–RUNX2, hypoxia for HIF-1α–SOX9, soft matrices for Wnt/β-catenin—and published in vitro and small-animal evidence shows attenuation rather than abolition of COL10A1 and MMP13 expression. Source-specific differences in baseline pathway activity likely underlie the frequently reported chondrogenic stability hierarchy (SM-MSC ≈ IFP-MSC > BM-MSC > ASC), although source ranking remains protocol- and species-dependent, and they argue for combinatorial suppression rather than single-pathway intervention as a basis for next-generation MSC products [43,44,45]. The molecular pathways governing MSC chondrogenic stability and hypertrophic drift are summarised in Figure 4.

Protective pathways including SOX9, HIF-1α and PTHrP promote stable hyaline cartilage formation through enhanced collagen II and aggrecan production. In contrast, activation of Wnt/β-catenin, RUNX2 and Indian Hedgehog (IHH) signalling promotes hypertrophic differentiation characterised by COL10A1 expression, calcification and endochondral ossification. The balance between these pathways ultimately determines regenerative success.

Beyond hypertrophy management, source selection is driven by accessibility. Adipose-derived stem cells (ASCs) are harvested at high yield from lipoaspirate with lower donor-site morbidity than iliac-crest bone-marrow harvest, making them attractive for combinatorial scaffold–cell strategies. Manferdini and colleagues showed that an RGD-functionalised injectable hydrogel (VitroGel^®^ RGD) supported ASC viability and chondrogenic commitment, with significant upregulation of SOX9, COL2A1 and ACAN versus non-functionalised controls—illustrating how ASC potential can be channelled toward hyaline-like output by deliberate scaffold design rather than soluble signals alone [46]. IFP-MSCs, frequently co-harvested at knee arthroscopy, combine accessibility with documented immunomodulatory capacity and represent an attractive autologous source for intra-articular delivery [47].

This accessibility is, however, balanced by an unresolved controversy regarding the functional state of IFP-MSCs harvested from the diseased joint, since cells exposed to the osteoarthritic pro-inflammatory milieu can be functionally “primed” in ways that affect their behaviour as cell-therapy products; this issue is treated in detail in Section 3.3.3 in the context of the IFP–synovium unit.

#### 3.1.3. Induced Pluripotent Stem Cells

Human iPSCs offer effectively unlimited expansion and patient-specific origin, addressing the donor-site and yield limitations of primary chondrocytes and MSCs [15]. Stable chondroprogenitor populations have been derived using developmentally inspired protocols and 3D aggregate culture, with reports of reduced hypertrophic propensity versus MSC-derived equivalents [48]. Allogeneic iPSC-derived cartilage organoids survived, integrated with native cartilage and contributed to repair without eliciting immune reactions in a cynomolgus monkey chondral-defect model—important preclinical proof-of-concept [49]. Translational hurdles remain prominent: genomic stability across passages, residual undifferentiated cells with tumourigenic potential, scalable GMP production, and the regulatory burden of a pluripotent-derived ATMP [50].

### 3.2. Scaffolds as Bioinstructive Microenvironments

Cartilage scaffolds are no longer designed solely to fill a defect and support cell adhesion. A bioinstructive scaffold concurrently delivers (i) a load-bearing template with bulk stiffness in the range of native cartilage and ideally depth-graded modulation, (ii) chondroinductive cues (TGF-β3, BMP-2, kartogenin, RGD or YIGSR peptides), (iii) a degradation profile matched to neotissue deposition over weeks to months, and (iv) tribological surface properties approaching the native friction coefficient (≈0.001–0.03) [51,52]. Surface functionalisation with lubricin (PRG4) or hyaluronic acid lowers hydrogel friction toward physiological values, reducing shear-induced apoptosis at the articulating surface and supporting the superficial-zone phenotype [23,51].

#### 3.2.1. Natural and Synthetic Hydrogels

Hydrogels remain the most extensively studied cartilage biomaterial because their hydrated three-dimensional networks resemble the cartilage ECM more closely than solid scaffolds [52,53]. Natural polymer hydrogels—hyaluronic acid (HA), collagen, gelatin, alginate, chitosan, silk fibroin—provide intrinsic biological recognition (e.g., HA–CD44 signalling supports chondrocyte phenotype and chondroprotection) but exhibit batch variability and limited tunable mechanics [54,55]. Synthetic hydrogel systems based on poly(ethylene glycol) (PEG) and poly(vinyl alcohol) (PVA) deliver reproducibility and tunable mechanics but lack inherent bioactivity; polycaprolactone (PCL), by contrast, is a hydrophobic biodegradable thermoplastic polyester used as scaffold framework, fibre or reinforcing phase, often combined with hydrogels for mechanical integrity. Composite and dynamically crosslinked hydrogels—HA-aldehyde/PEG-hydrazide and disulfide-crosslinked gelatin methacryloyl (GelMA)—combine these advantages, supporting injectability, self-healing and arthroscopic delivery [56,57]. GelMA has emerged as a workhorse cartilage bioink because its photocrosslinking allows fine spatial control while its gelatin backbone retains RGD motifs and MMP-cleavable sequences essential for cell-mediated remodelling [58].

#### 3.2.2. Decellularised Extracellular Matrix

Decellularised extracellular matrix (dECM) preserves the native chemical complexity—collagens, GAGs, fibronectin, growth-factor-binding domains—that even the most carefully designed synthetic systems cannot fully recapitulate [59]. Cartilage- and osteochondral-derived dECMs in solubilised, particulate or hybrid PCL-reinforced forms have shown chondroinductive activity in vitro and in small-animal models, and a recent meta-analysis of dECM-based therapies in OA reports favourable cartilage-repair scores compared with control treatments, although heterogeneity in decellularisation protocols, source tissue and cross-linking remains a significant limitation [60,61]. Light-activated dECM bioinks address mechanical-integrity concerns by introducing photocrosslinkable groups that improve construct stiffness without compromising the residual bioactivity of the matrix [62].

#### 3.2.3. D Bioprinting and Zonal Constructs

The osteochondral unit—superficial, middle and deep cartilage zones, calcified cartilage and subchondral bone—varies continuously in composition, mineral content and stiffness, and cannot be represented by a single homogeneous scaffold [63]. Three-dimensional bioprinting enables spatially controlled deposition of multiple bioinks, cells and signals, supporting multiphasic constructs that approximate native zonal organisation [64].

Among bioprinting modalities, digital light processing (DLP) uses a digital micromirror device to project a 2D image of each construct slice onto a vat of photocurable bioink, polymerising an entire layer in a single exposure. Because the build is nozzle-free, DLP avoids extrusion-related shear damage, achieves lateral resolution of 25–100 µm and supports zonally graded composition via sequential exposure of distinct bioinks [65,66]. Yang and colleagues used sequential DLP printing of collagen-based gradient bioinks to fabricate osteochondral scaffolds with continuous hyaline–calcified–bone transitions, supporting region-specific chondrogenic and osteogenic differentiation of BM-MSCs in vitro [67]. Long-term in vivo durability of such gradient constructs remains to be demonstrated.

#### 3.2.4. Stimuli-Responsive Scaffolds and Ultrasound-Activated Systems

Stimuli-responsive (“smart”) hydrogels alter swelling, stiffness, degradation or cargo-release in response to defined cues—pH, temperature, reactive oxygen species (ROS), enzymes, light, mechanical loading and ultrasound [68,69]. The OA joint provides a particularly tractable target because its microenvironment combines chronic inflammation, oxidative stress, MMP-rich matrix degradation and altered loading. Kuang and colleagues demonstrated a ROS-responsive hydrogel that scavenged ROS and delivered therapeutic payload locally, reducing inflammation and improving cartilage repair in a temporomandibular OA model [70]. Thermoresponsive injectable hydrogels exploit a sol–gel transition at body temperature for arthroscopic delivery into irregular defects [71].

Ultrasound has emerged as a particularly relevant stimulus because it is non-invasive, capable of focal tissue targeting and has clinical and regulatory precedent in selected bone-healing indications (notably non-union), though its role in cartilage tissue engineering remains investigational. Low-intensity pulsed ultrasound (LIPUS), typically delivered at 1–3 MHz, 30–200 mW·cm^−2^ (SATA) and 20% duty cycle, activates integrin-mediated mechanotransduction, ERK1/2 and PI3K/Akt signalling, upregulates SOX9, COL2A1 and ACAN, and inhibits IL-1β-driven NF-κB activation in chondrocytes [72,73]. In MSC chondrogenesis, LIPUS modulates autophagy, enhancing matrix deposition and exosome release [74]. Combined with ultrasound-responsive hydrogels containing piezoelectric microparticles or cavitation enhancers, LIPUS enables spatiotemporally controlled growth-factor release and on-demand stiffness modulation [75]. A 2024 review supports LIPUS as an investigational adjunct in tissue-engineered cartilage maturation, while emphasising the need for dose, frequency and timing standardisation before broad clinical adoption [76]. The case for stimuli-responsive systems must be tempered by limitations detailed in Section 4.2: limited penetration of short-wavelength NIR (700–900 nm, <1 cm), hyperthermia risk, GMP reproducibility, and the absence of a defined combination-product regulatory pathway.

### 3.3. Bioactive Signals and the Microenvironment

#### 3.3.1. Growth Factors and Spatiotemporal Delivery

TGF-β superfamily ligands (TGF-β1, TGF-β3, BMPs) drive mesenchymal condensation, chondrogenic differentiation and ECM synthesis [77]. Delivered as bolus injections, these pathways are highly context-dependent: prolonged or mistimed signalling drives hypertrophy, fibrosis or ectopic ossification rather than stable hyaline output [77,78]. Free growth-factor injection additionally suffers rapid clearance, dose-dependent off-target effects and short half-life [79]. Current strategies prioritise spatiotemporally controlled delivery: affinity-based platforms (heparin-mimetic peptides, decoy ECM domains), nanoparticle and microsphere carriers, and stimuli-responsive systems. Lee and colleagues showed that a crosslinked HA hydrogel encapsulating TGF-β3 produced sustained matrix synthesis and improved repair versus bolus delivery in a small-animal model [80].

#### 3.3.2. Paracrine and Cell-Free Strategies: Extracellular Vesicles and Progenitor-Recruiting Constructs

A substantial proportion of the therapeutic effect attributed to MSCs derives from their paracrine secretome rather than persistent engraftment [81]. MSC-derived small extracellular vesicles (sEVs) carry miRNAs (miR-92a-3p, miR-140, miR-100-5p), proteins and lipids that may modulate chondrocyte proliferation, ECM turnover and inflammation [81,82]; the term “exosome” should be reserved for vesicles with demonstrated endosomal origin in line with current MISEV reporting guidance. A 2025 review concluded that the majority of preclinical studies report beneficial effects of MSC-sEVs on cartilage matrix composition, chondrocyte apoptosis and matrix-degrading enzyme expression, while flagging consistent gaps in standardised potency, dose, biodistribution and regulatory classification [82]. In what follows, “cell-free” denotes strategies that do not deliver exogenous cells (relying instead on endogenous cell recruitment or paracrine signals such as EVs), whereas “acellular” refers to scaffolds or matrices processed to remove cells (e.g., dECM). Cell-free strategies reinterpret the cellular arm of the triad by relying on endogenous cell recruitment and paracrine modulation rather than ex vivo cell seeding: the construct delivers scaffold and signal components ex vivo, while host bone-marrow- and synovium-derived progenitors are recruited in situ via SDF-1/CXCR4 and MCP-1 gradients. These approaches fall within the broader in situ tissue engineering paradigm, in which the implant supplies scaffold and instructive cues rather than pre-seeded cells. Tu and colleagues demonstrated this principle with an injectable, self-healing exosome-crosslinked hydrogel that promoted hyaline-like neotissue formation and seamless osteochondral integration in vivo, supporting scaffold-assisted EV delivery as a translational route [83].

The translational case for MSC-derived EVs nevertheless requires deliberate critical reading, and five concrete obstacles remain unresolved. First, there is no consensus potency assay: no single miRNA cargo, surface marker or chondroprotective bioassay reliably predicts in vivo efficacy, and EVs from nominally identical sources differ according to donor age, passage and culture conditions. Second, GMP-compliant manufacturing is unsolved at scale; ultracentrifugation and size-exclusion chromatography dominate the preclinical literature but do not transfer cleanly to commercial volumes, and the available alternatives carry their own contaminant and yield trade-offs. Third, biodistribution after intra-articular injection is poorly characterised, leaving the proportion reaching cartilage versus cleared by synovium and lymphatics quantitatively unknown. Fourth, cargo control is incomplete: parental MSC heterogeneity propagates into EV cargo before any standardisation. Fifth, regulatory classification is unresolved—EVs sit at the intersection of biologics, ATMPs and combination products and are treated differently across jurisdictions, with no harmonised ICH framework specific to cartilage [82]. Until these are addressed, MSC-EV therapies should be regarded as a promising but preclinical technology rather than an imminent alternative to MACI or live-cell MSC products.

#### 3.3.3. Immune Responses to Pathologically Altered and Tissue-Engineered Cartilage

Cartilage was historically considered immunoprivileged because of its dense matrix and absence of vasculature. This view has been substantially revised: the OA joint is now understood as a low-grade inflammatory disease in which innate and adaptive immune signalling converge on cartilage destruction, and tissue-engineered constructs face a foreign-body response that materially affects their fate [84,85].

**Figure 5:**
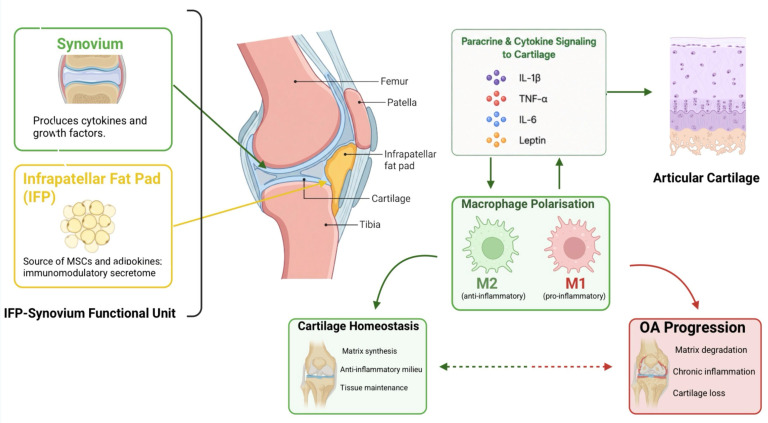
The infrapatellar fat pad–synovium functional unit in cartilage homeostasis and osteoarthritis.

In pathologically altered cartilage, mechanical and oxidative damage liberates damage-associated molecular patterns (DAMPs)—fibronectin fragments, hyaluronan oligosaccharides, HMGB1, S100A8/9 and aggrecan fragments—that signal through TLR2 and TLR4 on synovial macrophages and chondrocytes, activating NF-κB and inducing IL-1β, TNF-α, IL-6 and matrix-degrading enzymes (MMP-1, -3, -13; ADAMTS-4, -5) [86,87]. Synovial macrophages skew toward an M1 pro-inflammatory phenotype, amplifying matrix loss and chondrocyte apoptosis [88]. The infrapatellar fat pad (IFP) is no longer an inert space-filler: together with the synovial membrane, it undergoes adipocyte hypertrophy, fibrosis and CD68^+^ macrophage infiltration with ageing and obesity, releasing leptin, adipsin, IL-6, TNF-α and prostaglandins that act paracrinely on cartilage and synovium [89,90,91]. The integration of the fat pad and synovium into a single anatomo-functional unit and its implications for resident and donor-derived MSCs are well characterised [92,93,94]. The IFP–synovium unit therefore both confounds regenerative outcomes and constitutes a therapeutic target; conversely, IFP-MSCs are an accessible, chondrogenically competent autologous source co-harvested at arthroscopy [91]. The immunological interaction between the synovium, infrapatellar fat pad and cartilage is illustrated in Figure 5.

The infrapatellar fat pad and synovium form an integrated immunological and paracrine signalling unit that influences cartilage biology. Cytokines and adipokines including IL-1β, TNF-α, IL-6 and leptin regulate macrophage polarisation and can either support cartilage homeostasis or drive osteoarthritic progression. This microenvironment also affects the biological behaviour of resident and therapeutically harvested MSC populations.

The functional state of IFP-MSCs from the diseased joint remains controversial. Several studies indicate that IFP-MSCs exposed in vivo to the OA pro-inflammatory milieu are not phenotypically neutral but are functionally “primed” by surrounding cytokines, with altered CD10 and CD146 expression and a re-balanced immunoregulatory secretome [92]. Whether this priming is detrimental, neutral or beneficial remains debated: it can enhance immunoregulatory and analgesic actions—including CD10-mediated Substance P degradation and sustained antagonism of activated peripheral blood mononuclear cells [92]—but it also raises questions about chondrogenic fidelity, donor variability and reproducibility for cartilage repair. Donor joint status should therefore be treated as a potency-determining variable, and IFP-MSC products will require explicit characterisation of donor inflammatory context as part of release criteria. The same biology that licences IFP-MSCs as immunomodulators may complicate their use as primary chondrogenic effectors.

Tissue-engineered constructs encounter a related but distinct challenge—the foreign-body response. Within minutes of implantation, plasma proteins adsorb to the construct surface, providing ligands for monocyte adhesion and macrophage fusion into multinucleated giant cells; sustained M1 polarisation drives fibrous encapsulation, isolating the construct from native tissue [95,96]. Scaffold parameters with reproducible influence on immune trajectory include pore architecture (with specific pore-size ranges favouring M2 polarisation), surface chemistry (hydrophilic, low-zeta-potential surfaces tending to reduce pro-inflammatory signalling), endotoxin contamination (an underappreciated cause of inappropriate M1 activation) and degradation kinetics matched to neotissue deposition [96,97]. Allogeneic and xenogeneic dECM constructs additionally require attention to residual α-Gal epitopes and donor DAMP load. Immunomodulatory strategies include scaffold-bound IL-4 or IL-13, IDO-expressing carrier cells, biomaterial-bound TGF-β and co-delivery with MSCs whose paracrine activity favours M2 polarisation [85,98]. Xi and colleagues used single-cell transcriptomics to show that a dual-functional Si-A/PUE@HA hydrogel reduced pro-inflammatory macrophage signatures, enriched anti-inflammatory phenotypes and improved cartilage repair in an osteochondral defect model [99].

Comparative synthesis. Table 2 consolidates the main strategies discussed across cells, scaffolds and bioactive signals. The table is intended as an indicative overview rather than an exhaustive meta-analysis; specific quantitative benchmarks and references are developed in the body of each subsection.

### 3.4. Translational Maturity: Clinically Validated, Preclinical and Speculative Approaches

**Figure 6:**
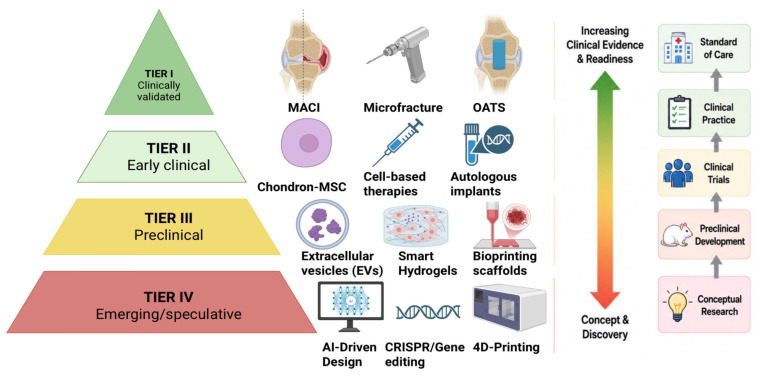
Translational maturity framework for cartilage tissue engineering technologies.

The strategies summarised in Table 2 occupy very different points on the translational spectrum, and conflating them risks giving equal weight to interventions with markedly different evidentiary standing. We therefore classify the principal approaches into four explicit tiers. The relative translational maturity of current cartilage regeneration strategies is summarised in Figure 6. Tier I—clinically validated approaches with regulatory approval and randomised long-term data—comprises microfracture for small focal defects, MACI (with two-, five- and ≥10-year comparative outcome evidence), and osteochondral autograft transfer; the SUMMIT trial and the ≥10-year MACI systematic review anchor this tier [15,35,36]. Tier II—approaches with first-in-human or early-phase clinical data but limited randomised long-term comparators—encompasses single-stage chondron–MSC co-implantation (the IMPACT trial and subsequent systematic review) [40,41] and low-intensity pulsed ultrasound (LIPUS), which has regulatory precedent in selected bone-healing indications but remains investigational for cartilage [76]. Tier III—preclinical and proof-of-concept technologies supported by in vitro and small-animal evidence—covers allogeneic iPSC-derived cartilage organoids in non-human primates [49], dECM scaffold-based constructs with encouraging preclinical meta-analytic evidence but limited and heterogeneous clinical data [60], stimuli-responsive (ROS-, thermo-, ultrasound-activated) hydrogels, MSC-derived extracellular vesicle products, immunomodulatory scaffolds, DLP- and extrusion-bioprinted zonal osteochondral constructs, and CRISPR-edited reparative cells (Section 3.2.3, Section 3.2.4, Section 3.3.2 and Section 4.1) [70,71,82,83,99]. Tier IV—speculative technologies with conceptual feasibility but sparse cartilage-specific in vivo or clinical evidence—includes 4D-printed shape-memory cartilage scaffolds, AI-generated zonal microarchitectures and closed-loop bioreactor maturation (Section 4). Tier assignments reflect the current evidentiary landscape and will require updating as higher-quality comparative and long-term data emerge; movement between tiers requires defined evidence (potency-assay standardisation, mechanistic in vivo demonstration, randomised comparison against MACI).

Cartilage repair strategies are stratified according to current clinical readiness. Tier I includes clinically validated approaches such as MACI, microfracture and osteochondral autograft transfer (OATS). Tier II comprises early clinical therapies including chondron–MSC constructs and cell-based interventions. Tier III encompasses preclinical technologies such as extracellular vesicles, smart hydrogels and bioprinted scaffolds, whereas Tier IV contains emerging concepts including AI-assisted scaffold design, CRISPR-mediated cell engineering and 4D printing. The right panel illustrates progression from conceptual development to standard clinical care.

## 4. Emerging Directions

### 4.1. CRISPR-Based Cell Engineering

CRISPR/Cas systems are increasingly applied not as direct in vivo therapies but as ex vivo platforms to engineer reparative cells before implantation [100,101]. Two strategies have gathered the strongest preclinical support. CRISPR/Cas9 knockout of MMP13 in primary human chondrocytes reduces matrix degradation and increases type II collagen accumulation, demonstrating that the catabolism–anabolism balance can be shifted by gene editing alone [102]. CRISPR-mediated transcriptional activation of SOX9 combined with inhibition of RELA enhanced the chondrogenic and immunomodulatory output of MSCs in an OA model, supporting the view that programmable transcriptional control may be more clinically attractive than simple gene knockout [103,104].

The translational distance between these proofs-of-concept and a regulatable cartilage product is, however, substantial, and a critical reading rests on four main obstacles. First, delivery: efficient editing of primary chondrocytes and MSCs without compromising chondrogenic phenotype remains technically demanding, as LNP and AAV vectors face cargo-size limits for Cas9 and bidirectional editors, while electroporation reduces vector immunogenicity but is poorly compatible with GMP-scale manufacturing. Second, fidelity: off-target editing is harder to exclude in primary human cells than in immortalised lines, and current best practice requires whole-genome sequencing with structural-variant analysis—a regulatory burden that adds rather than reduces cost. Third, mosaicism: edited cell populations are heterogeneous (wild-type, monoallelic and biallelic), with direct implications for batch potency and lot release. Fourth, regulatory standing: as of 2025, there are no approved CRISPR-based cartilage therapies. Exa-cel (Casgevy) provides a precedent for ex vivo CRISPR-edited haematopoietic products approved for sickle cell disease and transfusion-dependent β-thalassaemia, not for orthopaedic indications, and does not establish a pathway for scaffold-embedded chondrocyte or MSC products, since haematopoietic editing uses single-cell suspensions with controlled potency endpoints that do not generalise to a structural construct. CRISPRa and CRISPRi offer transcriptional modulation without necessarily altering DNA sequence, whereas base and prime editing reduce reliance on double-strand breaks but still introduce permanent genomic changes; both routes are only emerging in early clinical evaluation. CRISPR is therefore a platform under active development; integration into scaffold-based repair will require resolved safety, delivery, fidelity and phenotypic-stability data before claiming a defined translational role [100,101,104].

### 4.2. Advanced Biofabrication

Beyond DLP and extrusion bioprinting, emerging modalities widen the design space. Two-photon polymerisation enables sub-micrometre features for pericellular-matrix-scale cues; melt electrowriting deposits aligned poly(ε-caprolactone) (PCL) microfibres that, combined with hydrogel infiltration, produce composites whose stiffness, anisotropy and zonal organisation more closely approach native tissue than either component alone [105,106]. Bioink development has progressed from single-polymer formulations toward composite GelMA–methacrylated hyaluronic acid (HAMA), dECM-supplemented and nanocellulose-reinforced systems, with rheological and degradation profiles tuned for printability without compromising chondrogenic permissiveness [107]. Bioreactor maturation—physiologically relevant compression, shear and perfusion—integrated with non-invasive monitoring (Raman spectroscopy, optical coherence elastography) closes the development loop and shortens the design–validation cycle.

Four-dimensional (4D) printing extends static three-dimensional fabrication into the time domain by using shape-memory or stimuli-deformable materials whose geometry changes after deployment. Choudhury and colleagues fabricated a near-infrared (NIR)-responsive polylactide-co-trimethylene-carbonate scaffold nanoengineered with polydopamine that recovered >99% of its programmed shape within 30 s under low-power NIR (0.76 W·cm^−2^) and supported near-complete bone regeneration in critical-sized rabbit cranial defects, illustrating the principle of self-fitting deployment for irregular defects in bone tissue engineering [108]. For cartilage specifically, Deng and colleagues developed a mangrove-inspired bionic 4D-printed shape-memory polyurethane–nanohydroxyapatite composite that recovered its programmed geometry near body temperature, demonstrating conceptual feasibility of minimally invasive deployable cartilage scaffolds [109]. These advances are conceptually attractive but speculative for cartilage application: unresolved limitations include limited penetration of short-wavelength NIR (NIR-I, 700–900 nm; typically <1 cm in soft tissue), restricting triggerability of deeper osteochondral constructs; hyperthermia risk at shape-recovery temperatures; the absence of long-term in vivo durability data under continually loaded synovial conditions; and the lack of an established regulatory pathway for stimuli-responsive combination products in cartilage.

### 4.3. Artificial Intelligence and Data-Driven Approaches

Artificial intelligence (AI) and machine learning (ML) are increasingly applied to accelerate scaffold and process optimisation when the parameter space exceeds empirical iteration. In cartilage tissue engineering, these tools map onto trade-offs already enumerated: the printability–biocompatibility tension in 3D bioprinting (Table 2) and the multi-parameter tuning required to reinforce hydrogels without compromising chondrogenic output. Cartilage-specific demonstrations remain less mature than the broader tissue-engineering AI literature, but the optimisation problems are well-suited to ML. Demonstrated applications include prediction of bioink printability and post-print scaffold quality from rheology, nozzle geometry and ambient parameters [110], and broader ML frameworks supporting scaffold design, prediction of biological response, drug-delivery optimisation and image analysis [111]. Emerging directions, at earlier cartilage-specific evidence stages, have been proposed conceptually for zonal scaffold microarchitectures conditioned on target stiffness (generative or topology-optimised design), automated histological and MRI T2-map quantification, and closed-loop control of bioreactor regimens [112].

The optimistic case must nevertheless be tempered by three limitations. First, training-data quality and scale: cartilage-specific public datasets sufficient to train and externally validate predictive models across donor age, cell source, scaffold composition and outcome metrics are sparse and unstandardised, and single-laboratory models generalise poorly. Second, interpretability: regulatory acceptance of AI-derived design choices in ATMPs requires defensible, auditable rationales for individual predictions—deep models do not provide these by default, and explainable-AI overlays add engineering value but do not yet meet clinical-grade decision-support thresholds. Third, regulatory pathway: when AI tools are used to inform device geometry, lot release or implant selection, they fall within the FDA Software-as-a-Medical-Device (SaMD) and EU Medical Device Regulation frameworks, with requirements for data provenance, model freezing, post-market monitoring and change control that are not normally imposed on research-stage pipelines. AI accelerates defined optimisation steps within a workflow whose biological assumptions and regulatory accountabilities remain the investigators’ responsibility.

## 5. Conclusions

Cartilage tissue engineering has progressed from defect-filling to restoring depth-graded, mechanically competent and immunologically tolerant tissue. MACI remains a principal clinical benchmark with a 10-year benefit, but its two-stage logistics, dedifferentiation risk and cost have driven single-stage chondron- and progenitor-based therapies, ASC- and IFP-MSC-based approaches with deliberate hypertrophy suppression, and cell-free constructs that recruit endogenous progenitors. Scaffolds function as bioinstructive microenvironments, and the immunological dimension is now central to construct design. The four-tier translational maturity classification (Section 3.4) makes the distance to clinic explicit, and several specific challenges across cellular sources, construct design and emerging technologies must be solved.

At the cellular level, single-pathway suppression of hypertrophy (PTHrP, hypoxia, soft matrices) attenuates but does not abolish COL10A1 and MMP13 expression, and the next step will be combinatorial multi-node intervention engaging Wnt/β-catenin, IHH–PTHrP and HIF-1α in parallel, combined with cell sources of intrinsic chondrogenic stability such as SM-MSC and IFP-MSC and underpinned by standardised potency assays for objective batch release. Where IFP-MSCs are concerned, however, cells harvested from the diseased joint are not phenotypically neutral, and donor joint status should accordingly be formalised as a release variable, with prospective characterisation of the inflammatory secretome and clinical evaluation stratified by donor disease stage.

At the construct level, the foreign-body response and the IFP–synovium milieu are no longer peripheral concerns: immune-aware design—pore architecture and surface chemistry tuned for M2 polarisation, controlled endotoxin and α-Gal load, and integration of scaffold-bound IL-4/IL-13 or IDO-expressing cells—should be a built-in requirement of next-generation constructs rather than an optional refinement. Stimuli-responsive hydrogels (Tier III) and 4D-printed shape-memory scaffolds (Tier IV) likewise await resolution of NIR penetration depth, hyperthermia risk, GMP reproducibility and a defined combination-product regulatory pathway, and the actionable next steps are standardised stimulus dose, long-term in vivo durability data in continually loaded synovial environments, and early regulatory engagement before further proof-of-concept iteration.

At the emerging-technology frontier, the distance from MMP13 knockout or SOX9-activation proofs of concept to a regulatable CRISPR-based cartilage product turns on delivery, fidelity, mosaicism control, and a regulatory pathway distinct from the Casgevy haematological precedent, with programmable transcriptional control (CRISPRa/CRISPRi, base- and prime-editing) representing an attractive route that should be evaluated under the same scrutiny. The optimistic case for AI-guided design must in parallel be tempered by the absence of cartilage-specific datasets at scale, the interpretability deficit of deep models, and FDA Software-as-a-Medical-Device and EU MDR obligations; required next steps are shared cartilage-specific datasets with standardised outcome metrics, audit-grade explainable-AI overlays, and integration of AI as a defined optimisation step within validated workflows rather than as an unconstrained design generator.

Translation will therefore be paced not by single technical advances but by standardised potency assays, immune-aware construct design, disciplined long-term in vivo evaluation, and integration of these tools rather than their development in isolation.

## Figures and Tables

**Table 1:** Comparative analysis of principal cell sources in cartilage tissue engineering.

Cell source	Typical Use/Benchmark	Principal Strengths	Acknowledged Limitations
**Autologous chondrocytes**	Clinical benchmark (ACI/MACI) for symptomatic cartilage defects	Native cartilage phenotype; direct ECM production (collagen II, aggrecan); strong long-term clinical outcomes	Limited expansion capacity; dedifferentiation during monolayer culture; donor site morbidity; two-stage procedure and cost
**Mesenchymal stromal cells (MSCs)**	Widely used in regenerative constructs and early clinical trials	Multipotent; accessible from multiple tissues (bone marrow, adipose, synovium, infrapatellar fat pad); immunomodulatory; scalable	Hypertrophic differentiation (COL10A1, RUNX2 expression); fibrocartilage formation; donor variability; inconsistent long-term stability
**Induced pluripotent stem cells (iPSCs)**	Experimental/preclinical cartilage engineering	Unlimited expansion; patient-specific; high differentiation potential; reduced donor-site limitations	Tumourigenicity risk; genomic instability; residual undifferentiated cells; high regulatory and manufacturing complexity

**Table 2:** Comparative synthesis of major strategies in articular cartilage tissue engineering, with typical use, principal strengths and acknowledged limitations.

Approach	Typical Use/Benchmark	Principal Strengths	Acknowledged Limitations
**Microfracture**	Small focal defects, often <2 cm^2^	Single-stage, low cost, technically simple	Fibrocartilage with type I collagen predominance; declining benefit in some patients
**MACI (third-generation ACI)**	Established comparator for larger symptomatic defects	Best long-term clinical benchmark; 10-year outcome evidence	Two-stage procedure; expansion/dedifferentiation risk; cost
**Single-stage chondron/MSC therapies**	Selected focal lesions	Avoids two-stage expansion; pericellular matrix preserved in chondrons	Limited long-term randomised comparative data
**BM-MSC/ASC/SM-MSC/IFP-MSC**	Cell-source selection for regenerative constructs	Paracrine and immunomodulatory potential; autologous options	Hypertrophic drift; donor variability; potency-assay uncertainty
**Hydrogels (natural/synthetic/composite)**	Injectable or printable scaffold systems	Hydrated ECM-like environment; tunable crosslinking	Often mechanically weak unless reinforced or composite
**dECM scaffolds**	Biochemically rich scaffold environments	Preserve matrix-associated cues and growth-factor-binding domains	Protocol heterogeneity; residual immunogenicity; mechanical weakness
**3D bioprinting (DLP/extrusion)**	Zonal and osteochondral construct fabrication	Spatial control; gradient architecture; high resolution	Printability–biocompatibility trade-offs; limited long-term in vivo evidence
**Smart/LIPUS-responsive scaffolds**	On-demand signalling or mechanical stimulation	Non-invasive modulation; potential for controlled release and mechanotransduction	Dose, timing and standardisation remain unresolved
**MSC-derived extracellular vesicles**	Cell-free signalling strategy	Avoids live-cell engraftment issues; anti-inflammatory and pro-chondrogenic signals	Dose, potency, biodistribution and regulatory classification unresolved

## Data Availability

No new data were created or analysed in this study. Data sharing is not applicable to this article.
